# Characterization of dengue virus entry into HepG2 cells

**DOI:** 10.1186/1423-0127-16-17

**Published:** 2009-02-04

**Authors:** Lukkana Suksanpaisan, Tharinee Susantad, Duncan R Smith

**Affiliations:** 1 Molecular Pathology Laboratory, Institute of Molecular Biology and Genetics, Mahidol University, Salaya campus, 25/25 Phuttamontol Sai 4, Salaya, Nakorn Pathom, 73170, Thailand; 2 Molecular Medicine Program, Mayo Clinic College of Medicine, Guggenheim 18, 200 First street SW, Rochester, MN, 55905, USA; 3 National Science and Development Agency (NSTDA), 111 Thailand Science Park, Phahonyothin Road, Klong 1, Klong Luang, Pathumthani, 12120, Thailand

## Abstract

**Background:**

Despite infections by the dengue virus being a significant problem in tropical and sub-tropical countries, the mechanism by which the dengue virus enters into mammalian cells remains poorly described.

**Methods:**

A combination of biochemical inhibition, dominant negative transfection of Eps15 and siRNA mediated gene silencing was used to explore the entry mechanism of dengue into HepG2 cells.

**Results:**

Results were consistent with entry via multiple pathways, specifically via clathrin coated pit mediated endocytosis and macropinocytosis, with clathrin mediated endocytosis being the predominant pathway.

**Conclusion:**

We propose that entry of the dengue virus to mammalian cells can occur by multiple pathways, and this opens the possibility of the virus being directed to multiple cellular compartments. This would have significant implications in understanding the interaction of the dengue virus with the host cell machinery.

## Background

While most animal viruses enter into cells by receptor mediated endocytosis in clathrin coated pits [1,2], evidence to date suggests that the normal mechanism of dengue virus entry into both insect and mammalian cells is by direct fusion of the virus with the cell membrane [3-6], although endocytosis of dengue viruses has been observed with neutralization escape mutants [5], in the presence of neutralizing antibodies [6] as well as in the non-target cell line Hela [7]. These results are somewhat contradictory to results with other flaviviruses, and both Japanese encephalitis virus (JEV) and West Nile virus have been shown to enter cells via clathrin coated pits [8-10]. However, the majority of the studies undertaken to date on the dengue viruses have been based upon electron microscopy ultrastructural studies [4-6] or non-target cells [7] and as such no comprehensive direct biochemical or genetic analysis of the entry mechanism of the dengue virus has yet been undertaken.

## Methods

### Cells and viruses

HepG2 and Vero cells were maintained as previously described [11-13] Dengue serotypes 1 (strain 16007), 2 (strain 16681), 3 (strain 16562) and 4 (strain 1036) were propagated in Vero cells and purified as described previously [11,12].

### Cytotoxicity assessment by Annexin V staining

Confluent HepG2 cells were pretreated at 37°C with either 20 μM cytochalasin D or 30 μg/ml nystatin for 2 hr, or with 15 μg/ml chlorpromazine or 3 mM amiloride or 50 μM LY294002 or 0.2 μM wortmannin for 1.5 hr with 80% DMSO for 20 hours as a positive control. Cells were then trysinized and subsequently washed twice with cold-PBS. Cells were then washed with binding buffer (BD Biosciences Pharmingen, San Diego, CA) and resuspended in binding buffer. Annexin V-FITC (BD Biosciences Pharmingen, San Diego, CA) was added to the cell suspensions and samples were incubated in the dark for 15 min before analysis by flow cytometry (BD FACSCalibur *# *E6361).

### Biochemical inhibition of dengue entry

Confluent HepG2 cells were pretreated at 37°C with chlorpromazine, amiloride, wortmannin or LY294002 for 30 min or with cytochalasin D or nystatin for 1 hr. The cells were infected with each dengue serotype in either in the presence or absence of the appropriate inhibitor at an MOI of 1, for 1 hr at 37°C. The extracellular viruses were then inactivated by acid glycine (pH3) treatment [14]. The infected cells were further grown for one propagation cycle minus two hours with the exact time dependent upon the dengue serotype as determined previously [12], and the number of infected cells determined by our adaptation of the standard plaque assay [15]. Experiments were undertaken independently in triplicate with duplicate plaque assay of samples.

### Eps15 transfection, infection and indirect immunofluorescence microscopy

Plasmid constructs of dominant negative (DIII and EH29) and control (D3Δ2) Eps15 were kindly provided by A. Benmerah (Department of Infectious Diseases, Institut Cochin, Paris, France). Transfections of HepG2 cells were undertaken using lipofectamine2000 (Invitrogen, OR., USA). Briefly, 3 μg of the appropriate plasmid DNA was complexed with 4 μl of lipofectamine2000 for 30 min at room temperature and then added to HepG2 cells pre-grown to 70–80% confluency on glass coverslips. The cell/complex mix was incubated at 37°C, 10% CO_2 _for 2 days. Transfection efficiencies routinely exceeded 70% efficiency as determined by counting of multiple fields. Transfected cells were subsequently infected with dengue virus serotype 1, 2, 3 or 4 at an MOI of 20 for 1.5 hr followed by acid glycine (pH3) treatment to inactivate un-internalized viruses [14] and incubated for 15 hr at 37°C, 10% CO_2_. A further set of cells were serum starved for 30 min and incubated with 5 μg transferrin conjugated with Alexa 594 (Molecular Probes, OR) at 37°C for 30 min follow by acid glycine treatment.

Both dengue infected and transferrin treated transfected cells were fixed with 4% paraformaldehyde for 30 min at room temperature. Transferrin control cells were directly mounted with Vectashield (Vector Laboratories, Inc., CA) while dengue virus infected cells were further permeabilized with 0.3% TritonX-100 and methanol. Nonspecific binding was blocked by incubation with 5% BSA for 1 hr at room temperature. Cells were incubated with an anti-dengue E protein monoclonal antibody, HB-114 [16] at 4°C overnight. After six washes with 0.03% TritonX-100 in PBS cells were incubated with a chicken anti-mouse IgG conjugated with Alexa 594 (Molecular Probes, OR) for 1 hr at room temperature and subsequently washed with six washes of 0.03% TritonX-100 in PBS before mounting with Vectashield (Vector Laboratories, Inc., CA).

### siRNA design and generation

Target sites on clathrin heavy chain (GenBank accession number NM_004859) and the green fluorescent protein (GFP; GenBank accession number U50974) were determined using the on-line tool from Ambion, Austin, TX  and the selected sequences were subjected to siRNA template design to generate DNA oligonucleotide sequences for use with the Silencer™ siRNA Construction kit (Ambion). Six templates for siRNA generation were selected:

siCHC1: 298-AACCCAGCAACATTGGCTTC-318

siCHC2: 411-AAGTAATCCAATTCGAAGACC-431

siCHC3: 484-AAAGCTGGGAAAACTCTTCAG-504

siCHC4: 1951-AATAATCGCCCATCTGAAGGT-1971

siCHC5:3544-AATGAACCTGCGGTCTGGAGT-3564

siGFP: 295-AAAGATGACGGGAACTACAAG-315

Numbering indicates the corresponding position of the selected 21 nucleotide sequence in the open reading frame of NM_004859 (siCHC1 to siCHC5) or U50974 (siGFP). All sequences were searched against the NCBI's database to confirm specificity. Sense and antisense DNA templates were chemically synthesized (BioBasic, Canada) and following the kit instructions based on *in-vitro *transcription, the siRNAs were produced and quantified by spectrophotometry.

### siRNA transfection

HepG2 cells were maintained in Dulbecco's modified Eagle's medium (DMEM) supplemented with 10% heat-inactivated fetal bovine serum without antibiotics. Reverse transfections according to the manufacturers protocols were performed with Lipofectamine™RNAiMAX (Invitrogen, Carlsbad, CA) by mixing the respective siRNA and 1.2 μl of Lipofectamine™RNAiMAX and adding to a single well of a 24 well plate. After 20 minutes of incubation at room temperature, a suspension of 5 × 10^4 ^HepG2 cells was added and cell: complex mixtures incubated under standard conditions. Mock transfections (lipofectamine only) were performed in parallel. All transfections were undertaken in a final volume of 600 μl with siRNA at a final concentration at 50 nM. Transfections were harvested at 1 to 4 days post-transfection.

### RNA extraction and RT-PCR analysis

Transfected cells from a single well of a 24-well plate were homogenized in 0.5 ml Trizol reagent (Molecular Research Center, Cincinnati, OH) and RNA purified as recommended by the manufacturer. For the RT-PCR analysis, an oligo(dT)_17 _primer was used to synthesize first strand cDNA using ImpromII™ reverse transcriptase (Promega, Madison, WI). The cDNA was then amplified in a multiplex reaction with 2 specific primer pairs for CHC (CHCf: 5'-AAGCTCATCTTTGGGCAGAA-3'; CHCr: 5-GAGACAGCACCATCAGCAAA-3') and GAPDH (GAPDHf: TTGGTATCGTGGAAGGACTCA-3'; GAPDHr: 5'-ACCACCTGGTGCTCAGTGTAG-3') as an internal control. Expected products were 343 bp (GAPDH) and 222 bp (CHC) Cycle conditions were 94°C for 3 minutes followed by 20 cycles of 94°C for 30 seconds, 58°C for 45 seconds and 72°C for 45 seconds followed by a final extension of 72°C for 7 minutes. PCR products were analyzed on 1.8% agarose gels containing ethidium bromide.

### Infection of siRNA silenced HepG2 cells

HepG2 cells (5 × 10^4^) were grown on coverslips in single wells of a 24 well plate and transfected as above with either siRNAs as stated or mock transfected. At day 3 post transfection cells were infected with dengue virus serotype 2 MOI 20 for 2 hours followed by an acid glycine wash and subsequently incubated for 15 hours under standard conditions. Dengue virus E protein was detected as described above except that cells were also stained with DAPI. Parallel non-infected samples were incubated with transferrin as described above and were additionally stained with DAPI. Where biochemical inhibition was used in conjunction with siRNA silencing, samples were treated with 0.2 μM wortmannin for 30 minutes immediately preceding dengue virus infection.

## Results

### Effect of endocytosis inhibitors on dengue virus entry

**Figure 1:**
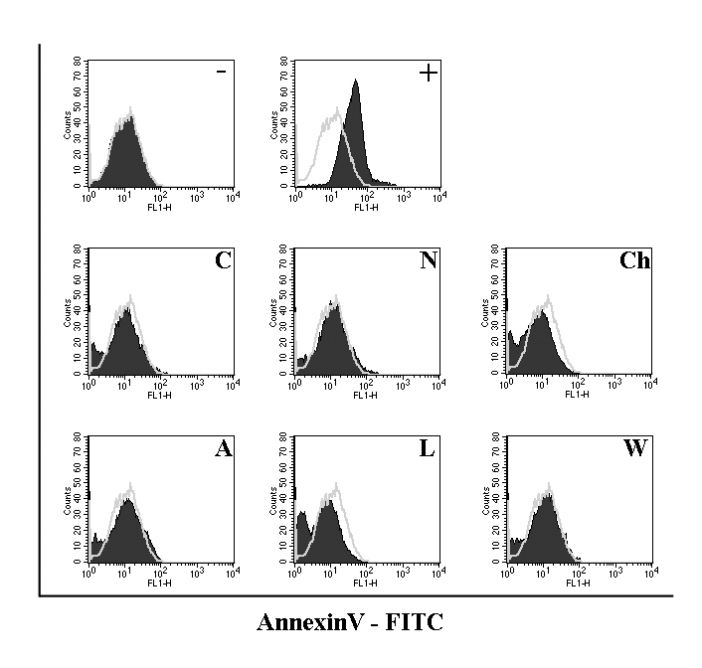
**Cytotoxicity assessement of biochemical inhibitors**. Flow cytometry histograms of HepG2 cells treated with working concentrations of cytochalasin D (C); amiloride (A); nystatin (N); chlorpromazine (Ch) LY294002 (L); wortmannin (W) or treated with 80% DMSO (+) or untreated (-).

To investigate the mechanism of dengue virus internalization into HepG2 cells, cells were pre-treated with cytochalasin D, amiloride, LY294002 or wortmannin to inhibit macropinocytosis, nystatin to inhibit caveolae mediated entry or chlorpromazine to inhibit clathrin-coated pit mediated. Prior to the infection experiment cells were incubated with a range of concentrations of the inhibitors to assess cytotoxicity. Cytotoxicity was initially assessed by cellular morphological changes under light microscopy (data not shown). Working concentrations (the highest concentration without apparent cytotoxicity) were established as: cytochalasin D at 20 μM, amiloride at 3 mM, LY294002 at 50 μM, wortmannin at 0.2 μM, nystatin 20 μM at 30 μM and chlorpromazine at 15 μM (HepG2). The lack of cytotoxicity at these concentrations was confirmed by Annexin V staining and flow cytometry (Figure 1).

To determine the effects of the various inhibitors on dengue virus entry, cells were pre-incubated for 1 hr with cytochalasin D or nystatin and for 30 min with chlorpromazine, amiloride, wortmannin or LY294002 at the concentrations determined above, following which the cells were incubated separately with all four dengue virus serotypes individually at an MOI of 1 for 1 hr following which the virus: cell mixtures were treated with acid glycine pH3 to inactivate any uninternalized viruses [14]. Cells were incubated under optimal growth conditions for a time equivalent to one virus replication cycle minus 2 hr based on our previous data for each serotype in HepG2 cells [12] following which the cells were briefly trypsinized, serially diluted and plated onto pre-grown cell monolayers and overlaid with agarose/nutrient medium as previously described for this adaptation of the standard plaque assay [15]. All experiments were undertaken independently in triplicate with duplicate assay of infected cell number.

**Figure 2:**
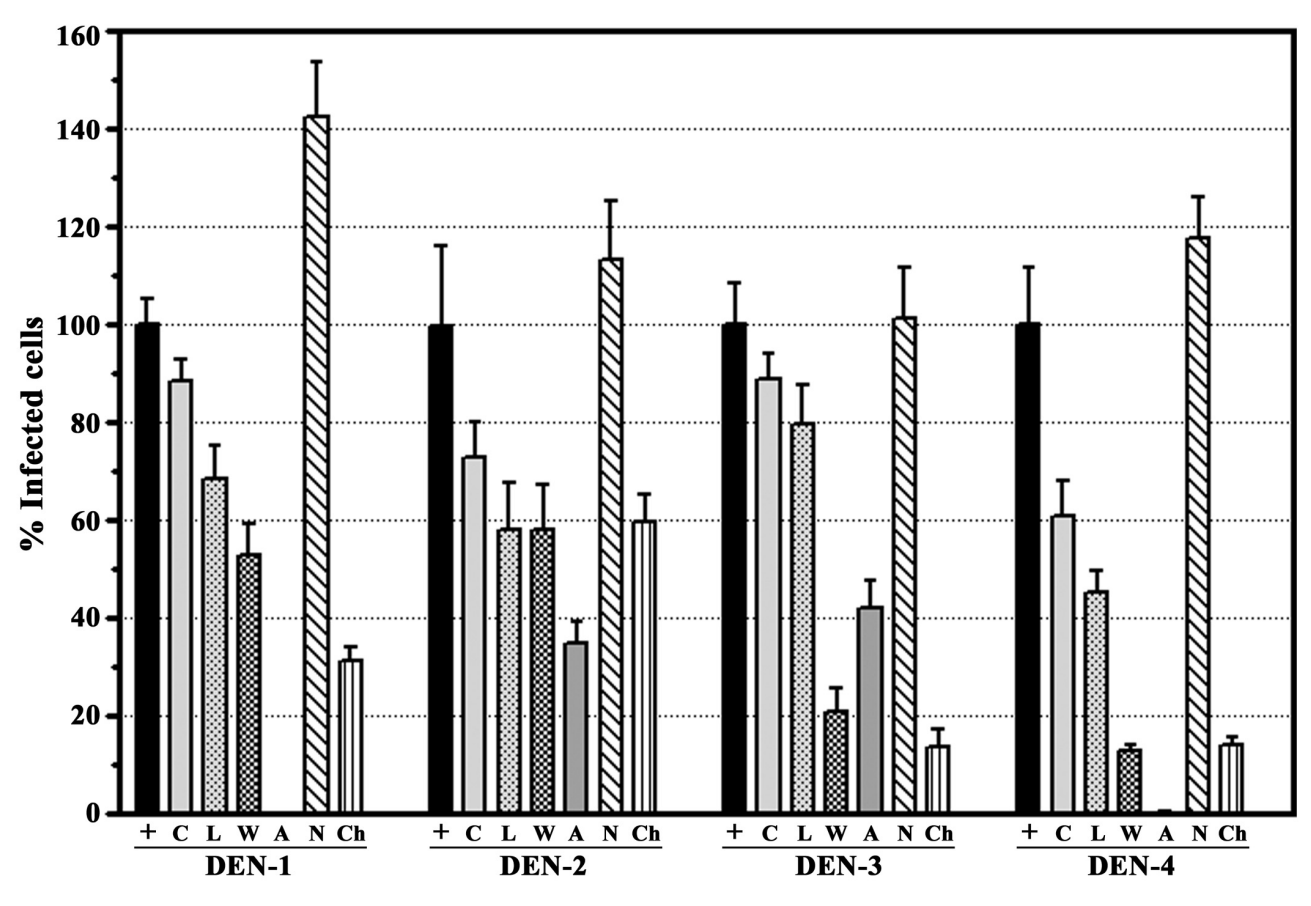
**Effects of biochemical inhibitors of endocytosis on dengue virus entry**. HepG2 cells were pre-incubated with cytochalasin D (C); amiloride (A); nystatin (N); chlorpromazine (Ch) LY294002 (L); wortmannin (W) or not pre-incubated (+) and subsequently infected with each serotype of the dengue viruses in the presence or absence of the respective treatment. Results are shown as a percentage of infected cells compared to control (100%). Error bar represent SEM of three independent experiments assayed in duplicate.

Results (Figure 2) show that inhibition of caveolae mediated endocytosis with nystatin results in a relatively uniform increase in the number of dengue virus infected cells for all serotypes, while inhibition of clathrin coated pit mediated endocytosis with chlorpromazine results in a significant reduction in the number of dengue infected cells for all four serotypes although the magnitude of the effect is variable. Inhibition of macropinocytosis with cytochalasin D, amiloride, LY294002 or wortmannin showed a broad range of effects depending upon the specific inhibitor used, as well as to some extent the serotype of the dengue virus (Figure 2).

**Figure 3:**
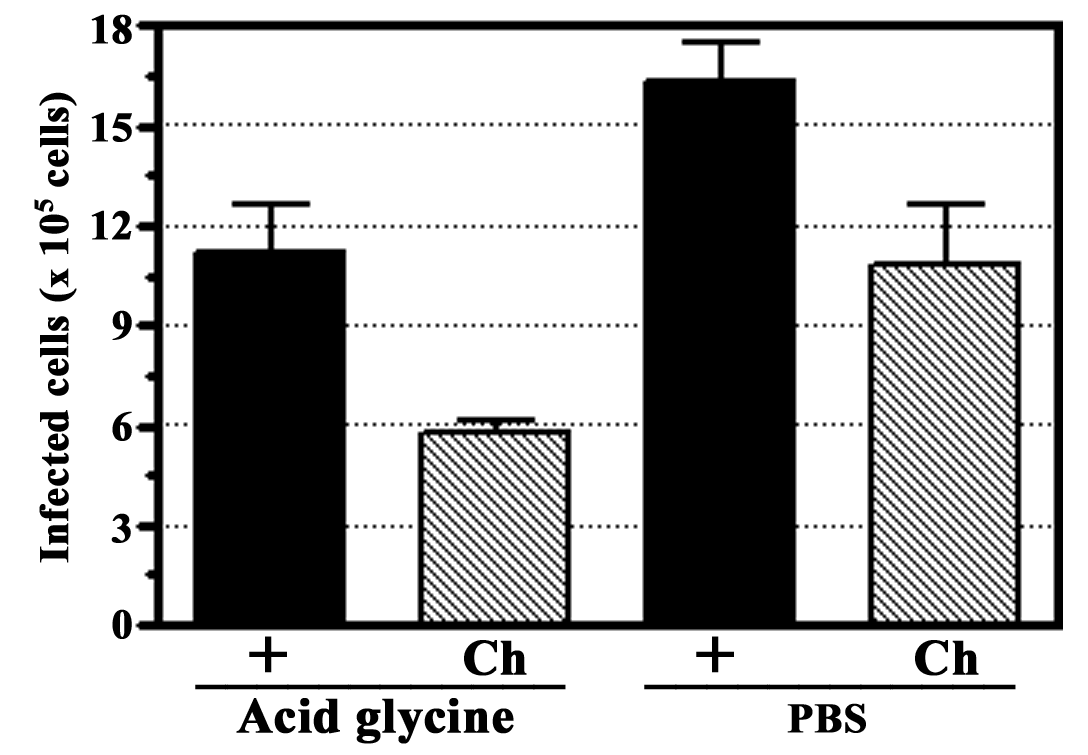
**Effect of acid glycine wash**. HepG2 cells were infected in the presence or absence of chlorpromazine (Ch) with our without an acid glycine wash. Results are shown as number of infected cells. Error bars represent SEM of three independent experiments assayed in duplicate.

Given that this study as well as our previous studies investigating dengue virus entry into HepG2 cells [11-13,15,17-20] have routinely employed acid glycine washes [14] to inactivate uninternalized viruses, we sought to determine whether this would materially affect the inhibition studies. The effect of acid glycine treatment was assessed by pre-incubating HepG2 cells with one the inhibitors (15 μM chlorpromazine) or with medium alone as a control. Cells were then infected with dengue virus serotype 2 at MOI of 1 in presence or absence of the inhibitor following which cells were either treated with acid glycine (pH3) or subjected to three washes with PBS. Numbers of infected cells were determined by our adaptation of standard plaque assay [15]. Results showed that washes with acid glycine did not increase virus entry due to promoting viral fusion at the cell surface (Figure 3).

### Dominant negative inhibition of clathrin coated pit endocytosis

Given that biochemical inhibitors can cause broad spectrum effects, we further sought to specifically knock out clathrin-dependent endocytosis using over-expressing dominant negative mutants of Eps-15 [21-23] which are able to effectively inhibit clathrin-mediated endoctyosis without affecting non-clathrin pathways [24]. HepG2 cells were transfected with either control (D3Δ2) or dominant negative mutants (DIII and EH29) of the Eps15 protein fused to GFP as well as the vector containing GFP only. Transfection with Lipofectamine2000 routinely resulted in transfection efficiencies of greater than 70% (data not shown). Transfected cultures were either infected with each of the four dengue virus serotype individually at MOI of 20, or incubated with Alexa 594 conjugated-transferrin before incubation and fixation. Visualization of signal was undertaken by incubating dengue infected samples with a primary monoclonal antibody directed against dengue E protein followed by incubation with a chicken anti-mouse IgG conjugated with Alexa 594.

**Figure 4:**
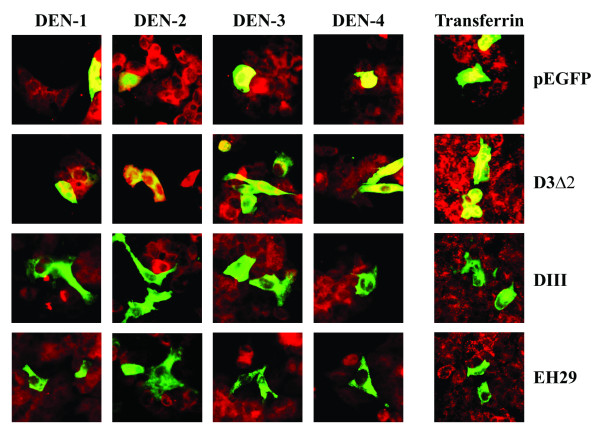
**Immunofluorescence of dengue infection and dominant negative mutant of Eps15**. HepG2 cell were transfected with either of two dominant negative Eps15 mutants (DIII or EH29) or a wild type Eps15 clone (D3Δ2) all fused to GFP or pEGFP as control, followed by infection with each dengue virus serotype individually or Alexa 594 conjugated-transferrin. Dengue infected samples were subsequently incubated with a mouse monoclonal antibody directed against dengue E protein and an Alexa 594 conjugated chicken anti-mouse IgG antibody. Signal from Alexa 594 (red) and GFP (green) were observed under a fluorescent microscope. Merged images are shown.

**Figure 5:**
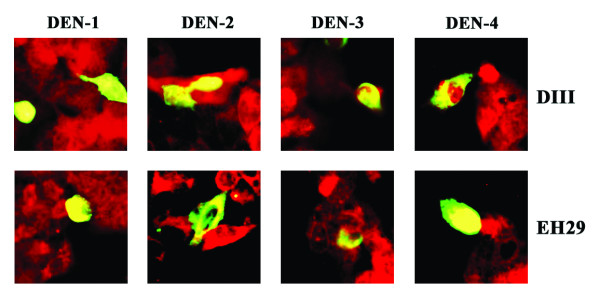
**Entry of the dengue virus in the presence of dominant negative mutants of Eps15**. Examples of cells positive for both dominant negative mutants of Eps15 (DIII or EH29)-GFP (green) and dengue virus infection (red). Merged images are shown.

Results show that both dominant negative Eps15 mutants (DIII and EH29) significantly excluded the entry of transferrin (Figure 4). However, while the two mutants predominantly excluded entry of all four dengue serotypes (Figure 4), numerous examples of dengue virus entry in the presence of expression of the dominant negative mutants were observed (Figure 5). Quantitation by counting multiple fields (n > 30) suggested that 15- to 20% of cells expressing either of the mutants were positive for dengue virus entry for each serotype. In contrast, only scattered cells were seen to be potentially positive for transferrin entry in the presence of the mutants, and these were possibly due to cells overlaying each other.

### siRNA mediated inhibition of clathrin heavy chain expression

Given the significant entry of the dengue virus in the presence of over-expressing dominant negative mutants of Eps15, it is possible that either entry was occurring via multiple pathways, or the Eps15 mutants were not completely inhibiting clathrin mediated entry. To further explore this, RNA interference was used to down regulate the expression of clathrin heavy chain, an integral part of the clathrin vesicle [25]. Five different siRNAs (siCHCs) against human clathrin heavy chain (GenBank accession number NM_004859) were generated using in vitro transcription together with 1 siRNA targeted to the green fluorescent protein (GFP; GenBank accession number U50974) for use as a control. To confirm all siRNAs were double-stranded, an aliquot of each siRNA was treated with RNaseIII which digests double-stranded RNA or RNaseA which digests single-stranded RNA. All siRNA constructs were confirmed to be off the appropriate size and to consist of dsRNA (data not shown).

To optimize the silencing of the expression of the clathrin heavy chain, the 5 different siCHCs were transfected into HepG2 cells in parallel with transfections of siGFP and lipofectamine alone (mock). On days 1 to 4 days post-transfection, cells were harvested and RNA extracted. Multiplex RT-PCR was undertaken to detect messages from GAPDH and clathrin heavy chain (CHC) simultaneously and results analyzed by agarose gel electrophoresis. Experiments were undertaken independently in triplicate.

**Figure 6:**
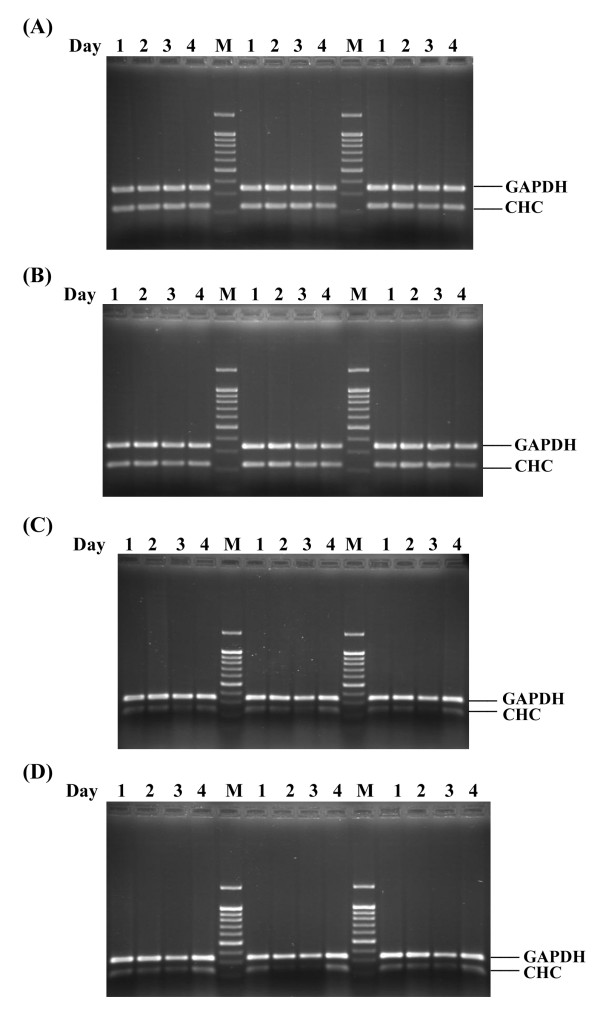
**Silencing of Clathrin heavy chain in HepG2 cells**. Multiplex RT-PCR products for GAPDH or clathrin heavy chain (CHC) of HepG2 cells either mock transfected (A); transfected with siGFP (B); transfected with siCHC3 (C) or transfected with siCHC5 (D). Samples represent day 1 to 4 post transfection and transfections were undertaken independently in triplicate.

Results showed a constant signal for GAPDH and the clathrin heavy chain (CHC) for mock and siGFP transfection (Figure 6). Message for clathrin heavy chain was seen to be significantly reduced for all transfections, with the greatest signal reduction being seen on day 3 post infection and for siCHC3 and siCHC5 (Figure 6).

### Dengue virus serotype 2 infection of clathrin heavy chain silenced HepG2 cells

Optimal silencing of clathrin heavy chain expression was noted at day 3 post transfection with siRNA constructs siCHC3 and siCHC5. These two siRNAs were again transfected into HepG2 cells as above in parallel with transfections of siGFP and mock (transfection agent only) and on day 3 post transfection cells were infected with dengue virus serotype 2 and an MOI of 20 and cells allowed to grow for 15 hours (the time for one replication cycle of dengue serotype 2 minus two hours) under optimal conditions. At 15 hours cells were either analyzed by microscopy or by our adaptation of the standard plaque assay [15] to determine the number of infected cells. Both microscopy and determination of infected cell number were undertaken independently in triplicate.

**Figure 7:**
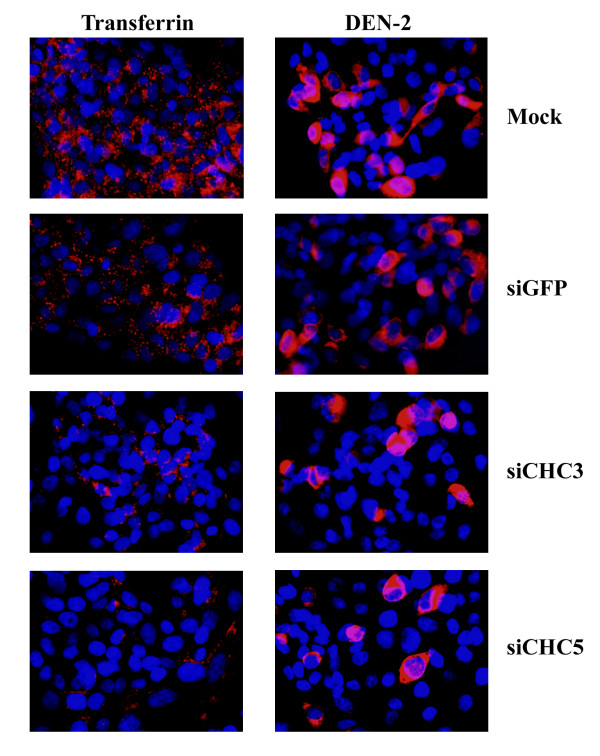
**Dengue serotype 2 infection of siRNA silenced HepG2 cells**. Indirect immunoflourescence of HepG2 cells either mock transfected or transfected with siGFP, siCHC3 or siCHC5 and subsequently either infected with the dengue virus (red) or incubated with transferrin (red). Cells were additionally stained with DAPI (blue) to show nuclei. Merged images are shown.

Consistent with our results with transfections of dominant negative constructs of Eps15, a significant reduction of transferrin entry was seen with siCHC transfections, but not with mock or siGFP (Figure 7). Dengue virus serotype 2 entry was observed for mock and siGFP transfections (Figure 7). While transfections of siCHC constructs again reduced dengue virus entry significantly, entry of the virus was still observed. Levels of entry of the dengue virus serotype 2 in siCHC transfected cells was again observed to be on the order of 15 to 20% of cells as determined by counting multiple microscope fields (n > 30).

**Figure 8:**
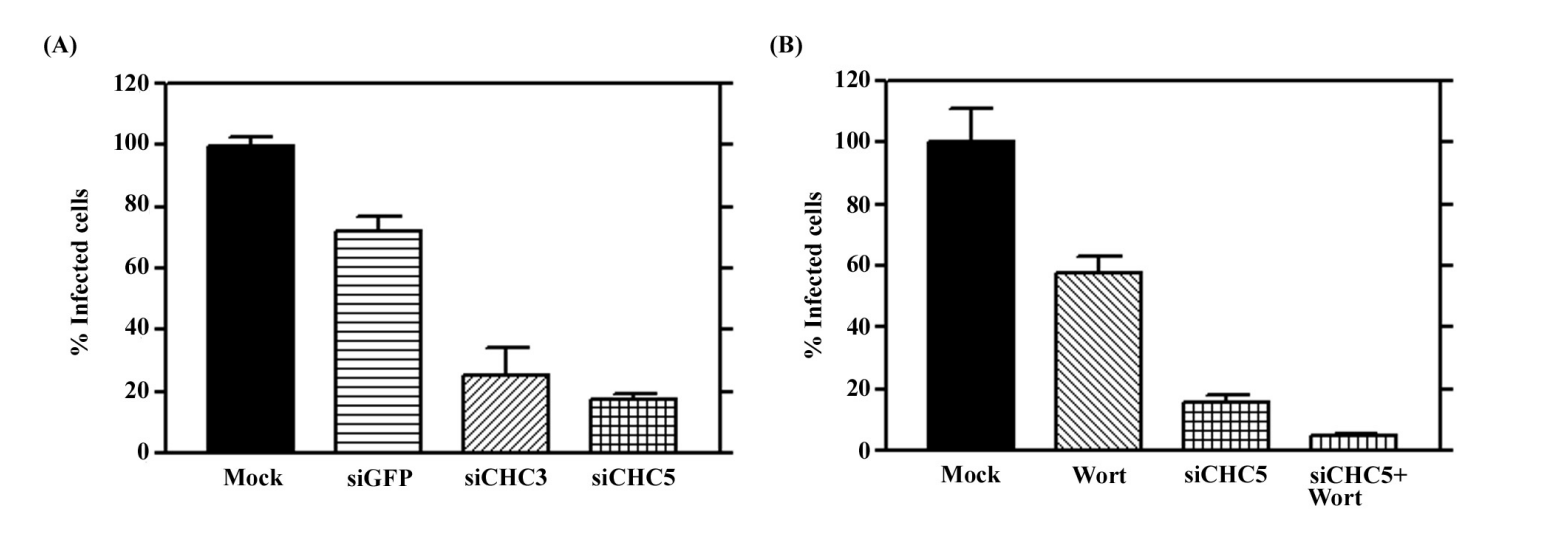
**Infection of siRNA silenced HepG2 cells**. A. HepG2 cells either mock transfected (mock) or transfected with siGFP, siCHC3 or siCHC5 were infected with dengue virus serotype 2 at MOI 20 and the number of infected cells determined by our adaptation of the standard plaque assay [15]. Results are expressed as a percentage of mock infection and error bars represent the SEM of three independent experiments assayed in duplicate. B. HepG2 cells were either mock transfected or transfected with siCHC5 and either treated or not treated with wortmannin prior to infection with the dengue virus serotype 2 at MOI 20. Results are expressed as a percentage of mock infection and error bars represent the SEM of three independent experiments assayed in duplicate.

Results of dengue virus entry as seen by our adaptation of the standard plaque assay [15] were consistent with the results observed by microscopy. We observed a slight reduction of dengue virus entry in siGFP transfected cells as compared to wild type (mock transfected) suggesting that transfection of even irrelevant siRNAs may marginally affect the viability of the HepG2 cells. A significant reduction in the number of dengue virus infected cells was observed with the siCHC transfections (Figure 8, Panel A), with the number of infected cells again being some 20% of wild type (mock transfected) cells.

### Simultaneous inhibition of macropinocytosis and clathrin dependent endocytosis

To determine whether the approximately 20% virus entry seen in cells in which clathrin mediated endocytosis has been inhibited is a result of background, or the result of dengue virus entry by macropinocytosis, we sought to simultaneously inhibit both pathways, clathrin mediated endocytosis through siRNA mediated RNA inhibition and macropinocytosis through biochemical inhibition using wortmannin.

Cells were therefore transfected with siCHC5 to silence clathrin heavy chain or mock transfected and on day 3 post-transfection were either treated or not treated with wortmannin for 1 hour before being either incubated with transferrin or infected with dengue serotype 2 at an MOI of 20. Following acid glycine treatment of dengue infected cells, cells were incubated for 15 hours before being either examined by microscopy or the number of infected cells determined by our adaptation of the standard plaque assay [15]. Experiments were all undertaken independently in triplicate.

**Figure 9:**
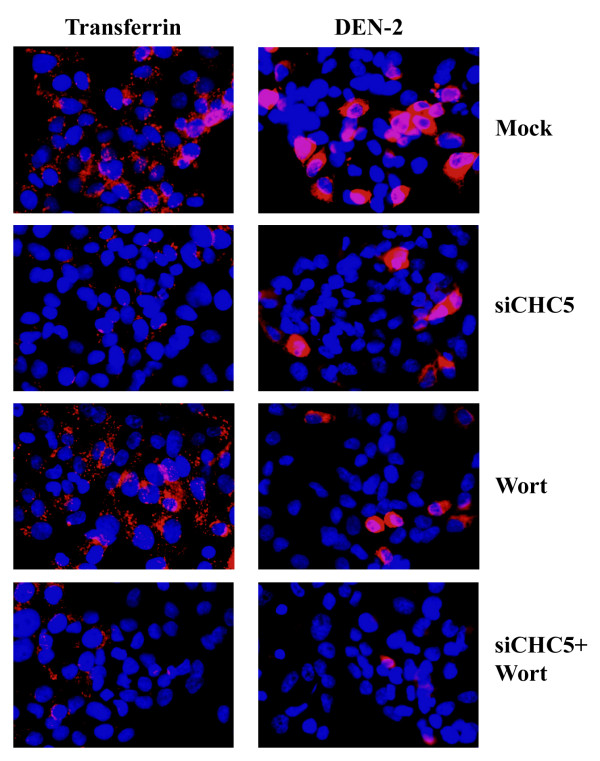
**Simultaneous inhibition of macropinocytosis and clathrin mediated endocytosis in HepG2 cells and infection with dengue virus serotype 2**. Indirect immunoflourescence of HepG2 cells either mock transfected or transfected with siCHC5 and subsequently either treated or not treated with wortmannin prior to infection with the dengue virus (red) at MOI 20 or incubated with transferrin (red). Cells were additionally stained with DAPI (blue) to show nuclei. Merged images are shown.

Results (Figure 9) show that transfection with siCHC5, but not treatment with wortmannin significantly excluded transferrin and a similar level of transferrin exclusion was seen between cells treated with siCHC5 alone and cells treated with siCHC5 and wortmannin in combination. HepG2 cells treated with either siCHC5 or wortmannin both showed a reduction in levels of dengue infected cells as compared to control, but a significant number of dengue infected cells was observed in each case. The combination of siCHC5 and wortmannin treatment resulted in the infection of rare, single scattered cells (Figure 8, Panel B).

Results from our adaptation of the plaque assay (Figure 7, Panel B) provided consistent data, with a significant reduction in the number of dengue serotype infected cells seen in the cells treated with a combination of siCHC5 transfection and wortmannin as compared to the siCHC5 transfected cells alone.

## Discussion

Despite flaviviral infections representing a significant world wide public health threat, little advance has been made in dissecting out the mechanism by which flaviviruses enter into either mammalian or insect cells. Studies on Japanese encephalitis virus and West Nile virus with either Vero (African Green monkey cells) or C6/36 cells have suggested that these two viruses enter by clathrin coated pit mediated endocytosis [8-10]. With the dengue virus however, data to date, which has been predominantly generated through electron microscopy studies [4-6], has suggested that direct fusion with the plasma membrane is the standard mode of entry of the dengue viruses.

Recently Chu et al., [9] provided evidence that West Nile virus enters into Vero cells via clathrin mediated endocytosis [9]. The authors however noted that pre-treatment of Vero cells with cytochalasin D (an inhibitor of macropinocytosis) resulted in an inhibition of infection and the authors proposed that this was possibly due to an effect upon virus trafficking due to cytochalasin D mediated de-polymerization of actin filaments [9]. In light of the results seen here it is possible that West Nile virus also enters via multiple pathways and the reduction seen in West Nile virus entry in the presence of cytochalasin D is a consequence of ablation of the macropinocytosis pathway rather than a consequence of altering virus trafficking. Further support for this is seen that reduction of West Nile virus entry in the presence of an Eps15 dominant negative mutant is some 80%, giving some 20% virus entry in the presence of the dominant negative mutant [9] – a figure comparable with the data presented here for the dengue virus.

Interestingly Chu and colleagues also investigated the entry of West Nile Virus into the aedes albopictus cell line C6/36 using the same dominant negative mutant of Eps15 [8] and similarly saw 15 to 20% entry of the virus in the presence of the mutant suggesting that West Nile virus may similarly enter into cells of both an insect and a mammalian origin by multiple pathways.

More recently Krishnan and colleagues have investigated the entry of the dengue virus into HeLa cells [7]. This study also used dominant negative mutants of Eps15 to ablate clathrin mediated endocytosis, and similarly concluded that the dengue virus entered by clathrin coated pit mediated endocytosis. Similar to Chu and Ng [9] however, some 20% virus entry as compared to wild type levels was observed, again giving the possibility that alternate pathways are responsible for the entry of some dengue virus into cells, and indeed, all four studies, this study and those of Chu and Ng [9], Chu and colleagues [8] and Krishnan and colleagues [7] suggest that ablation of clathrin coated pit mediated endocytosis only reduces virus entry by 80%.

Our data suggests that the remaining 20% virus entry observed is not the results of incomplete ablation of clathrin mediated endocytosis but represents virus entry by a viable, independent pathway, macropinocytosis. Entry of the dengue viruses (and more possibly flaviviruses in general) by multiple pathways as shown here raises some interesting questions, particularly with respect to the initial flavivirus: host cell interaction and may require a significant re-evaluation of our understanding of flavivirus entry into host cells.

## Conclusion

Consistently, inhibition of clathrin mediated endocytosis using dominant negative mutants of Eps 15 results in a reduction of dengue virus entry of approximately 80% as shown by this study and others [7-9]. Our data shows that the incomplete ablation of virus entry is not a result of incomplete knock down of clathrin mediated endocytosis, but rather reflects entry via an alternate pathway.

## Competing interests

The authors declare that they have no competing interests.

## Authors' contributions

LS undertook the biochemical inhibition studies and Eps15 transfections and analyzed and interpreted the data. TS undertook the siRNA mediated silencing studies and analyzed and interpreted the data. DRS was responsible for design and implementation of the study as well as drafting the manuscript. All authors read and approved the final version.
